# The Association Between Media Use for Parenting Information (MUPI) and Parenting Knowledge, Attitudes and Practices Among Parents of Children Aged 6–19 Years

**DOI:** 10.1111/cch.70233

**Published:** 2026-01-29

**Authors:** Pornnapasorn Nguansiri, Komsan Kiatrungrit, Sirichai Hongsanguansri, Nitchawan Jongrakthanakij, Wanlop Atsariyasing, Vilawan Chirdkiatgumchai, Chosita Pavasuthipaisit

**Affiliations:** ^1^ Child, Adolescent, and Family Psychology, Faculty of Medicine Ramathibodi Hospital, Faculty of Medicine Siriraj Hospital, and National Institute for Child and Family Development Mahidol University Bangkok Thailand; ^2^ Department of Psychiatry, Faculty of Medicine Ramathibodi Hospital Mahidol University Bangkok Thailand; ^3^ Department of Psychiatry, Faculty of Medicine Siriraj Hospital Mahidol University Bangkok Thailand; ^4^ Department of Pediatrics, Faculty of Medicine Ramathibodi Hospital Mahidol University Bangkok Thailand; ^5^ Institute of Child and Adolescent Mental Health Bureau of Mental Health Academic Affairs Bangkok Thailand

**Keywords:** health information, media use, parenting attitudes, parenting knowledge, parenting practices

## Abstract

**Background:**

The rise of digital media has significantly altered how parents access information regarding child development and parenting. While such platforms provide accessible support, the influence of different media formats on parenting outcomes remains underexplored. This study aimed to (1) assess the frequency of media use for parenting information (MUPI) and (2) examine its associations with parenting knowledge, attitudes and practices among Thai parents of children aged 6–19 years.

**Methods:**

A cross‐sectional study was conducted with 445 parents recruited through social media and in‐person outreach at hospitals and schools. Participants completed four validated self‐report instruments: the MUPI questionnaire, the Child and Adolescent parenting Knowledge Evaluation (CAKE), the Parenting Sense of Competence Scale (PSOC) and the Alabama Parenting Questionnaire (APQ). Descriptive statistics, Kendall's tau‐*b* correlations and multivariate linear regression analyses were employed to explore the associations.

**Results:**

Reading online materials was the most frequent form of media use and was positively associated with higher parenting knowledge, parental involvement and positive parenting practices. In contrast, frequent consumption of short‐form video content was linked to lower parenting knowledge, parental monitoring and greater inconsistency in discipline practices.

**Conclusion:**

Media activity matters. Engagement with evidence‐based, text‐based resources and structured online trainings appear to support better parenting outcomes, while overreliance on short‐form video content may be detrimental. These findings underscore the need for media literacy promotion and the development of accessible, high‐quality digital parenting content tailored to diverse parental needs.

## Summary

- Different types of digital media are associated with distinct parenting outcomes; reading online materials and participating in structured online training are linked to higher parenting knowledge and positive practices.
- Short‐form video content, while popular, is associated with lower parenting knowledge and more inconsistent discipline, highlighting the need for critical evaluation of such content.
- Thai parents show a strong preference for parenting content produced by health professionals, emphasising the importance of trusted, evidence‐based sources.
- Parenting applications and government websites are underutilised, suggesting the need for more user‐friendly, interactive and regularly updated platforms.
- These findings support media literacy promotion and development of tailored, high‐quality digital parenting resources as part of broader parenting support strategies.

## 1 Introduction

Parenting is a critical determinant of children's developmental outcomes, serving as a central protective factor that promotes resilience, and mitigates the impact of adversity (Ryan et al. 2017; Sanders and Turner 2018). It is a complex, dynamic process shaped by the interrelated domains of knowledge, attitudes and practices (National Academies of Sciences et al. 2016). Parenting knowledge refers to the information that parents acquire through formal education or personal experience; attitudes encompass their beliefs, values and perceptions about parenting, while practices are the behaviours and strategies employed in childrearing. However, even when parents have access to accurate information, applying that knowledge into daily parenting can be challenging. Barriers such as conflicting personal beliefs, limited parental self‐efficacy and contextual stressors often hinder the translation of knowledge into effective practices (Baku et al. 2017; Vale‐Dias and Nobre‐Lima 2018).

In today's digital world, media has become an increasingly influential source of parenting information. Beyond traditional sources such as healthcare professionals and family members, parents now turn to diverse media platforms, including websites, social media and mobile applications, to seek guidance and support in raising their children (Fierloos et al. 2022; Frey et al. 2022; Setyastuti et al. 2019). A growing body of literature highlights the potential of media use for parenting information (MUPI) to enhance parental knowledge, increase self‐efficacy and improve emotional well‐being through mechanisms such as reassurance, normalisation and empowerment (Chua and Shorey 2022; Frey et al. 2022; Kostyrka‐Allchorne et al. 2022). Despite these supportive evidence, existing research has largely concentrated on some media categories and Western populations, often focusing on parents of young children. As a result, the applicability of these insights to diverse cultural contexts and developmental stages remains limited (Kubb and Foran 2020; Sullivan et al. 2021; Vale‐Dias and Nobre‐Lima 2018).

To address these gaps, the present study focuses on Thai parents of children aged 6 to 19 years, examining the role of media in shaping parenting across middle childhood and adolescence. Specifically, this study aims to (1) assess the frequency of MUPI and (2) explore the associations between MUPI and key parenting domains, including knowledge, attitudes and practices.

## 2 Methods

### 2.1 Participants and Procedure

This cross‐sectional study included 445 Thai parents or primary caregivers of children aged 6–19 years. Participants were recruited using convenience sampling through both online platforms (Facebook, Instagram, LINE) and offline settings (a public hospital and three schools in Bangkok). Including both online and in‐person recruitment aimed to enhance sample diversity, reaching parents with varying levels of digital literacy, socioeconomic status and access to technology.

Eligible participants were Thai‐speaking adults (≥ 18 years) who were the primary caregiver of at least one child aged 6–19 years. Informed consent was obtained from all participants before enrolment. The required sample size was calculated using Cochran's formula for proportion estimation, assuming a 95% confidence level and a 5% margin of error. The minimum sample size was determined to be 385, with an adjusted target of 424 to account for potential nonresponses. Upon completion of the study instruments, participants received access to a complimentary digital parenting guidebook developed by the authors, titled A Guide to Child and Adolescent Care. Ethical approval for the study was granted by the Institutional Review Board (Approval No. MURA2024/380).

### 2.2 Measures

#### 2.2.1 Parent–Child Demographic Survey

The demographic survey includes seven items on parent information: sex (male, female), age, education (high school diploma or less, bachelor's degree, higher than bachelor's degree), residential region (Bangkok Metropolitan, Central, Northern, Southern, Northeastern, Eastern, Western, or abroad), employment status (yes/no), perceived financial burden (yes/no) and partnership status (living with a partner: yes/no). In addition, six items capture child‐related information: number of children, age, sex (male only, female only, both male and female), whether any child identifies as LGBTQ+ (yes/no), family structure (nuclear, extended, cross‐generational) and health conditions (developmental delays, chronic mental health, chronic physical health, or multiple health conditions).

#### 2.2.2 MUPI

The MUPI is a self‐report questionnaire developed by the authors to assess preferred sources of parenting information (government, hospitals, professionals and influencers) and the frequency of eight media activities: reading printed or online materials, watching short or long videos, listening to audio content, participating in online interactive trainings, asking questions on social media and using parenting apps for obtaining parenting information. Responses are rated on a 5‐point scale (0 = *never* to 4 = *5–7 days/week*), yielding total scores from 0 to 32. The instrument demonstrated excellent content validity (S‐CVI/Ave = 1.00) and modest internal consistency (*α* = 0.63). The modest alpha likely reflects usage patterns in Thailand, where parenting apps and online trainings are rarely used, producing floor effects and reduced variance, whereas printed materials, online sources and short videos are more frequently endorsed (see Questionnaire S1).

#### 2.2.3 Child and Adolescent parenting Knowledge Evaluation (CAKE)

The CAKE is a self‐report parenting knowledge assessment developed by the authors to evaluate knowledge across four domains: child development, health and safety, effective discipline and media mediation, covering the developmental period from middle childhood through adolescence. The scale includes 20 multiple‐choice items, each with five response options and one correct answer, yielding total scores from 0 to 20. The instrument demonstrated excellent content validity (S‐CVI/Ave = 0.93) based on expert review and was piloted with 10 caregivers of children aged 6–19 years, whose feedback informed revisions to the final version. The scale also showed good internal consistency (Cronbach's *α* = 0.70; see Questionnaire S2).

#### 2.2.4 Parenting Sense of Competence Scale (PSOC)‐Thai Version

The 17‐item self‐report instrument, originally developed by Gibaud‐Wallston and Wandersman (1978) and translated into Thai by Suwansujarid et al. (2013), assesses parents' perceived competence through two subscales: (1) skill/knowledge (eight items) and (2) valuing/comfort (nine items). Participants respond using a 6‐point Likert scale, with negatively worded items reverse scored. Total scores range from 17 to 102, where higher scores indicate greater perceived parenting competence. The Thai version of the questionnaire demonstrated good internal consistency, with a Cronbach's alpha of 0.78.

#### 2.2.5 Alabama Parenting Questionnaire (APQ)‐Thai Version

The 42‐item self‐report tool, originally developed by Frick (1991) and translated into Thai by Khamon et al. (2019), evaluates six parenting dimensions: (1) positive parenting, (2) parental involvement, (3) poor monitoring/supervision, (4) inconsistent discipline, (5) corporal punishment and (6) other discipline. Items are rated on a 5‐point Likert scale (1 = *never* to 5 = *always*). The Thai version has demonstrated good internal consistency, with Cronbach's alpha values exceeding 0.70 across all domains.

### 2.3 Data Analysis

Descriptive statistics, including frequencies, means and standard deviations, were used to summarise participant demographics, MUPI and parenting outcomes. To examine associations between demographic characteristics and parenting variables, Kendall's tau‐*b* correlation coefficients were used. Kendall's tau‐*b* was chosen for its robustness in handling data that is not normally distributed and for its ability to assess associations without assuming linearity or equal intervals between the categories of the variables. Multiple linear regression analyses were conducted to examine the relationships between media use types and parenting knowledge, attitudes and practices. Both unadjusted and adjusted models were reported, with adjustments made for relevant participant characteristics. In addition, subgroup analyses were performed to compare parents of school‐age children (6–11 years) and parents of adolescents (12–19 years).

## 3 Results

### 3.1 Participant Flow and Sample Size

**FIGURE 1:**
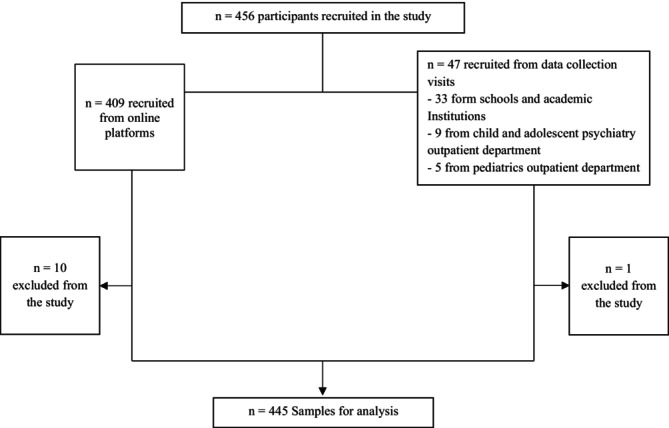
Flowchart of participant recruitment and inclusion. A total of 456 participants were initially recruited into the study. Of these, 409 were recruited through online platforms and 47 were recruited via in‐person data collection visits (33 from schools and academic institutions, 9 from child and adolescent psychiatry outpatient departments and 5 from paediatrics outpatient departments). Eleven participants were excluded due to ineligibility or incomplete responses, resulting in a final analytical sample of 445 participants.

As illustrated in Figure 1, participants were recruited through both online platforms and on‐site visits at schools and hospitals. Of the 456 participants initially enrolled, 409 were recruited online and 47 via in‐person channels. Eleven participants were excluded due to missing data or failure to meet inclusion criteria, resulting in a total of 445 participants included in the final analysis.

### 3.2 Participant and Child Characteristics

As shown in Table 1, among the 445 participating parents, the mean age was 42.71 years (*SD* = 6.19), with the majority identifying as female (91.68%). Most participants were middle‐aged (71.68%), employed (77.75%), in partnered relationships (83.59%) and living in nuclear family structures (53.25%). Almost half (49.88%) reported having more than one child. Regarding child characteristics, 37.75% of participants had only daughters and 32.13% indicated that their child had at least one chronic health condition.

**TABLE 1:** Characteristics of study participants and their child (*n* = 445).

Parameters		*n* (%)
Parental characteristics		
Parental sex	Male	36 (8.08)
	Female	408 (91.68)
	LGBTQ+	1 (0.22)
Parental age	Young adult (20–39 years)	120 (26.96)
	Middle age (40–59 years)	319 (71.68)
	Senior adult (over 60 years)	6 (1.34)
Parent education	High school diploma or less than	31 (6.96)
	Bachelor's degree	235 (52.80)
	Higher than bachelor's degree	179 (40.22)
Residential region	Bangkok metropolitan region	278 (62.47)
	Central region	33 (7.41)
	Northern region	43 (9.66)
	Southern region	17 (3.73)
	Northeastern region	33 (7.41)
	Eastern region	31 (6.96)
	Western Region	9 (2.02)
	Abroad	1 (0.22)
Employment	Unemployed	96 (21.57)
	Employed	346 (77.75)
Financially burdened	Yes	70 (15.73)
	No	375 (84.26)
Living in partnerships	Yes	372 (83.59)
	No	73 (16.40)
Child characteristics		
Number of children	Single child	223 (50.11)
	Multiple children	222 (49.88)
Sex of child	Only male	164 (36.85)
	Only female	168 (37.75)
	Both male and female	113 (25.39)
Age of child	School‐age children (6–12 years)	262 (58.87)
	Teenager (13 years and above)	183 (41.12)
Family structure	Nuclear	237 (53.25)
	Extended	201 (45.16)
	Cross‐generational	7 (1.57)
Availability of LGBTQ+ child	Yes	9 (2.02)
	No	436 (97.97)
Child with health problems	Yes	143 (32.13)
	Developmental delays	10 (2.24)
	Chronic mental health	64 (14.38)
	Chronic physical health	53 (11.91)
	Multiple health conditions	16 (3.59)
	No	302 (67.86)

### 3.3 Frequency of MUPI

As presented in Table 2, the most frequently reported MUPI activity was reading online materials (*M* = 2.81, *SD* = 1.22), followed by watching short video clips (*M* = 1.65, *SD* = 1.57) and long video clips (*M* = 1.51, *SD* = 1.26). The least frequently used resource was parenting applications (*M* = 0.18, *SD* = 0.58). Independent samples *t*‐tests revealed significant group differences between parents of school‐age children and parents of teenagers. Parents of school‐age children reported greater use of printed materials (*t* = 2.17, *p* = 0.030) and online materials (*t* = 3.48, *p* < 0.001; see Table S2).

**TABLE 2:** Frequency of media use for parenting information (MUPI) among parents (*n* = 445).

	OM	SV	LV	PM	OL	QO	OT	PA
Never, *n* (%)	19 (4.26%)	159 (35.73%)	109 (24.49%)	188 (42.24%)	237 (53.35%)	304 (68.31%)	350 (78.65%)	393 (88.31%)
1–4 days/month, *n* (%)	70 (15.73%)	89 (20.00%)	156 (35.05%)	124 (27.86%)	90 (20.22%)	82 (18.42%)	81 (18.20%)	35 (7.86%)
5–7 days/month, *n* (%)	59 (13.25%)	31 (6.96%)	61 (13.70%)	42 (9.43%)	36 (8.08%)	17 (3.82%)	8 (1.79%)	8 (1.79%)
1–4 days/week, *n* (%)	127 (28.53%)	80 (17.97%)	84 (18.87%)	65 (14.60%)	51 (11.46%)	25 (5.61%)	6 (1.34%)	6 (1.34%)
5–7 days/week, *n* (%)	170 (38.20%)	86 (19.32%)	35 (7.86%)	26 (5.84%)	31 (6.96%)	17 (3.82%)	0 (0.00%)	3 (0.67%)
Mean (SD)	2.81 (1.22)	1.65 (1.57)	1.51 (1.26)	1.14 (1.27)	0.98 (1.30)	0.58 (1.06)	0.25 (0.55)	0.18 (0.58)
Min–max	0–4	0–4	0–4	0–4	0–4	0–4	0–3	0–4

Abbreviations: LV, long video clips; OL, online listening; OM, online materials; OT, online interactive training; PA, parenting application; PM, printed materials; QO, questions on social media; SV, short video clips.

### 3.4 Parenting Knowledge, Attitudes and Practices

As shown in Table 3, parents demonstrated relatively strong parenting knowledge, with a mean score of 15.5 (*SD* = 3.12) out of 20. Parenting attitudes, as measured by the PSOC, were moderate (*M* = 71, *SD* = 10.5; range = 17–102). In terms of practices, positive parenting behaviours were prevalent (*M* = 25.6, *SD* = 3.13) and parental involvement was high (*M* = 39.4, *SD* = 4.88). In contrast, negative practices were reported less frequently, including poor parental monitoring (*M* = 15.7, *SD* = 4.97), inconsistent discipline (*M* = 14.0, *SD* = 3.63) and corporal punishment (*M* = 4.68, *SD* = 1.82). Independent samples *t*‐tests revealed that parents of school‐age children reported significantly higher levels of positive parenting practices (*t* = 3.09, *p* = 0.002) and parental involvement (*t* = 3.52, *p* < 0.001) compared with parents of teenagers. Conversely, parents of teenagers reported significantly higher levels of poor parental monitoring and supervision (*t* = −6.48, *p* < 0.001; see Table S2).

**TABLE 3:** Summary of parenting knowledge, attitudes and practices (*n* = 445).

Parameters		Min–max (points)	*M* (*SD*) (points)	Cut off score (points)
Parenting knowledge (CAKE)		2–20	15.51 (3.11)	—
Parenting attitudes (PSOC)		37–102	71.02 (10.49)	—
Parenting practices (APQ)	Positive parenting practices	15–30	25.63 (3.13)	≥ 21
	Parental involvement	17–50	39.40 (4.88)	≥ 35
	Poor parental monitoring/supervision	10–35	15.66 (4.96)	≥ 18
	Inconsistent discipline	6–26	14.01 (3.62)	≥ 18
	Corporal punishment	3–12	4.68 (1.82)	≥ 7

Abbreviations: APQ, Alabama Parenting Questionnaire; CAKE, Child and Adolescent parenting Knowledge Evaluation; PSOC, Parenting Sense of Competence Scale.

### 3.5 Linear Regression Analysis of MUPI on Parenting Outcomes

Table 4 presents the unadjusted (Model 1) and adjusted (Model 2) regression coefficients (*β*) and 95% confidence intervals examining the associations between different types of media use and parenting knowledge, attitudes and practices.

**TABLE 4:** Linear regression analysis of media use for parenting information (MUPI) on parenting knowledge, attitudes, and practices.

	Parenting knowledge	Parenting attitudes	Parenting practices				
	CAKE	PSOC	PP	PI	PPM	ID	CP
	*β* (95% CI)	*β* (95% CI)	*β* (95% CI)	*β* (95% CI)	*β* (95% CI)	*β* (95% CI)	*β* (95% CI)
Model 1							
PM	0.15 (−0.07–0.38)	1.35 (0.57–2.14)***	0.13 (−0.10–0.36)	0.43 (0.07–0.80)*	−0.23 (−0.60–0.13)	−0.15 (−0.42–0.12)	−0.06 (4.51–5.38)
OM	0.49 (0.25–0.74)***	0.48 (−0.35–1.33)	0.42 (0.17–0.68)**	0.71 (0.32–1.10)***	−0.66 (−1.06–0.26)**	0.05 (−0.34–0.24)	−0.12 (−0.26–0.02)
SV	−0.40 (−0.59 to −0.21)***	−0.61 (−1.2–0.02)	−0.13 (−0.32–0.06)	−0.19 (−0.49–0.10)	0.51 (0.20–0.81)**	0.40 (0.18–0.63)***	0.11 (−0.00–0.22)
OT	0.49 (−0.02–1.01)	1.12 (−0.65–2.89)	−0.15 (−0.68–0.38)	0.12 (−0.69–0.94)	0.86 (0.03–1.70)*	−0.36 (−0.97–0.25)***	−0.17 (−0.48–0.13)
*R* ^2^	0.07	0.04	0.03	0.05	0.04	0.03	0.01
Model 2							
PM	0.18 (−0.04–0.40)	1.34 (0.55–2.13)***	0.09 (−0.13–0.33)	0.39 (0.03–0.75)*	−0.05 (−0.41–0.29)	−0.15 (−0.43–0.11)	−0.06 (−0.19–0.07)
OM	0.38 (0.13–0.63)**	0.44 (−0.42–1.32)	0.35 (0.09–0.61)**	0.49 (0.09–0.89)*	−0.38 (−0.78–0.09)	−0.07 (−0.38–0.22)	−0.09 (−0.25–0.05)
SV	−0.30 (−0.49 to −0.12)**	−0.56 (−1.22–0.09)	−0.09 (−0.28–0.10)	−0.05 (−0.35–0.23)	0.34 (0.04–0.63)*	0.39 (0.17–0.62)***	0.07 (−0.03–0.19)
OT	0.54 (0.02–1.06)*	1.38 (−0.43–3.21)	−0.03(−0.57–0.14)	0.37 (−0.45–1.21)	0.43 (−0.39–1.25)	−0.42 (−1.06–0.21)	−0.27 (0.59–0.04)
*R* ^2^	0.17	0.11	0.12	0.15	0.19	0.10	0.09

*Note:* Model 1 represents the regression analysis without controlled characteristics of study participants. Model 2 represents the regression analysis with controlled characteristics of study participants, including parent age, education, employment status, partnerships status, family structure, financial burden, number of children, child age, having a child with LGBTQ+, and having a child with health problems.

Abbreviations: CAKE, Child and Adolescent parenting Knowledge Evaluation; CP, corporal punishment; ID, inconsistent discipline'; OM, online materials; OT, online interactive training; PM, printed materials; PI, parental involvement; PP, positive parenting practices; PPM, poor parental monitoring/supervision; PSOC, parenting sense of competence; SV, short videos.

* *p* < 0.05.

** *p* < 0.01.

*** *p* < 0.001.

In Model 1, reading online materials was significantly associated with higher parenting knowledge (*β* = 0.49, *p* < 0.001), positive parenting practices (*β* = 0.42, *p* = 0.001) and greater parental involvement (*β* = 0.71, *p* < 0.001) and was negatively associated with poor parental monitoring (*β* = −0.66, *p* = 0.001). Similarly, printed materials were positively associated with parenting attitudes (*β* = 1.35, *p* < 0.001) and parental involvement (*β* = 0.43, *p* = 0.018). In contrast, frequent use of short video clips was negatively associated with parenting knowledge (*β* = −0.40, *p* < 0.001) and positively associated with poor parental monitoring (*β* = 0.51, *p* = 0.001) and inconsistent discipline (*β* = 0.40, *p* < 0.001).

Model 2, which controlled characteristics of study participants, including parent age, education, employment status, partnerships status, family structure, financial burden, number of children, child age, having a child with LGBTQ+ and having a child with health problems, revealed consistent patterns of association, with an improved model fit (higher *R* ^2^ value). Online reading materials remained positively associated with parenting knowledge (*β* = 0.38, *p* = 0.003), positive parenting practices (*β* = 0.35, *p* = 0.008) and parental involvement (*β* = 0.49, *p* < 0.001). Printed materials continued to be significantly associated with positive parenting attitudes (*β* = 1.34, *p* = 0.015) and involvement (*β* = 0.39, *p* = 0.031). Additionally, the use of online training was positively associated with parenting knowledge (*β* = 0.54, *p* = 0.040). Conversely, frequent use of short video clips remained negatively associated with parenting knowledge (*β* = −0.30, *p* = 0.001) and positively associated with poor parental monitoring/supervision (*β* = 0.34, *p* = 0.023) and inconsistent discipline (*β* = 0.39, *p* < 0.001).

Separate regression analyses for parents of school‐age children and parents of teenagers indicated age‐specific patterns of association. Among parents of school‐age children, watching long video clips was negatively associated with parenting attitudes (*β* = −1.24, *p* = 0.042) and positively associated with poor parental monitoring (*β* = 0.53, *p* = 0.031). Asking questions on social media was positively associated with parental involvement (*β* = 0.75, *p* = 0.018) and corporal punishment (*β* = 0.25, *p* = 0.042). For parents of teenagers, the use of short videos (*β* = 0.53, *p* = 0.013) and asking questions on social media (*β* = −0.80, *p* = 0.008) were significantly associated with inconsistent discipline (see Table S3).

## 4 Discussion

This study examined the frequency of MUPI among Thai parents of children ages 6–19 years and to explore the association between different media formats and parenting knowledge, attitudes and practices. The findings indicate that parents primarily sought parenting information through online reading resources, followed by short video and long video formats. Notably, engagement with both online and printed materials and participation in online training were associated with higher parenting knowledge, attitudes and practices. In contrast, more frequent use of short‐form video content was associated with lower parenting knowledge and less consistent parenting practices.

### 4.1 MUPI

Consistent with prior studies, parents favoured accessible, flexible formats such as websites, social media platforms and e‐books over mobile apps or structured online training (C. S. Mott Children's Hospital University of Michigan Health 2023; Mertens et al. 2024). However, the effectiveness of these formats largely hinges on parents' ability to critically evaluate the quality and credibility of the information they find (Harris and Jacobs 2023; Kubb and Foran 2020). Professional guidance remains essential to help parents critically navigate digital content and make informed decisions about child development and care (Onishi 2024).

The increasing popularity of short‐form video platforms, such as TikTok, Instagram Reels and YouTube Shorts, is also reflected in the current findings (Puridej 2025; Settachan 2021). Short videos deliver parenting content in engaging, bite‐sized formats through visual storytelling and brief text summaries, enabling rapid sharing and peer learning (Liu et al. 2023; Rugrien 2022; Xiao et al. 2023). Additionally, content creation has expanded beyond professionals to include parents themselves, broadening the availability of both expert‐driven and experience‐based parenting advice (Anisa et al. 2023; Archaphet et al. 2021; Moungchinda 2022).

Despite these strengths, parenting apps had relatively low engagement, likely due to limited awareness, usability challenges and a lack of content that addresses the diverse needs of parents. Many existing apps cater primarily to expectant or first‐time parents and offer general advice or tracking tools (Rosawan Areemit et al. 2020; R. Areemit et al. 2023; David et al. 2024). In contrast, parents of older children may find these tools less relevant or difficult to integrate into daily life. Improving the effectiveness of parenting apps may require better user‐friendly interfaces, broader content coverage across developmental stages and evidence‐based content tailored to diverse parenting needs (David et al. 2024).

Parents in this study demonstrated a strong preference for content developed by health professionals (83.60%), as shown in Table S1, reinforcing recent findings that expert‐driven guidance remains the most trusted and actionable source for parents (Mause et al. 2022; Strehlke et al. 2025). In contrast, government websites were rarely selected as preferred sources, likely due to usability barriers, irregular updates or limited interactivity (Kessara et al. 2024; Wang and Inkuer 2024). These findings point to a need for institutional digital platforms to be more responsive to users' expectations and preferences.

Subgroup analyses further revealed that parents of school‐age children reported significantly greater use of printed and online materials compared with parents of adolescents. This pattern aligns with evidence that parents of younger children continue to value tangible and structured resources such as books and educational websites, which are perceived as reliable for supporting early learning and health guidance (Mertens et al. 2024). In contrast, as children enter adolescence, parents may increasingly rely on peer networks, school communications or social media platforms for information (C. S. Mott Children's Hospital University of Michigan Health 2023). These findings underscore the importance of tailoring digital parenting interventions to the child's developmental stage, with more structured and text‐based resources for parents of younger children and more interactive, socially embedded content for parents of adolescents.

### 4.2 MUPI and Parenting Knowledge, Attitudes and Practices

Our study found that greater engagement with text‐based materials, both in print and online, was associated with higher levels of parenting knowledge, attitudes and practices. Engagement with textual content typically involves deeper cognitive processing, which has been linked to better comprehension and information retention in prior research (El Sherif et al. 2023). This pattern may reflect that parents who engage more extensively with text‐based resources tend to hold more accurate beliefs about child development and report greater consistency in parenting practices.

Participation in interactive online training was also associated with higher parenting knowledge, consistent with prior research (Reyes et al. 2024). Online training programs often combined direct instruction with interactive learning elements such as Q&A sessions, role‐playing and shared reflections, which may support learning and application of parenting strategies (Reyes et al. 2024; Yamanaka et al. 2023). These features may help explain the observed association between online training participation and more favourable parenting outcomes.

Conversely, frequent use of short‐form video content was associated with lower parenting knowledge and less consistent parenting practices, such as reduced monitoring and inconsistent discipline. Although short videos are engaging and accessible, their brevity may limit critical engagement with complex parenting topics (Rugrien 2022; Zhang et al. 2022). Prior studies have reported associations between excessive short video use and reduced attention functions (Yan et al. 2024), which may partly help explain the observed relationship. However, it is also possible that preexisting attentional difficulties influence both media preferences and parenting behaviours. Parents with attention regulation challenges may struggle to maintain consistent caregiving and may prefer short‐form content due to its brevity and stimulation (Chen et al. 2023). This suggests that both the cognitive effects of media use and individual differences in attention may contribute to variations in parenting practices.

Separate regression analysis revealed that asking questions on social media was associated with both greater parental involvement and increased use of corporal punishment among parents of school‐age children. Parents may turn to online platforms when facing childrearing challenges, seeking experiential knowledge, social support and second opinions. While peers and professionals' support can be associated with greater parental confidence and involvement (Frey et al. 2022; Yamashita et al. 2022), exposure to diverse social norms may also coincide with greater acceptance of corporal punishment, particularly in sociocultural contexts where physical discipline remains normative (Duong et al. 2020; Taylor et al. 2011). These findings underscore the importance of professionally moderated platforms that emphasise evidence‐based guidance and discourage harmful practices (Taylor et al. 2016).

### 4.3 Strengths and Limitations

This study has several notable strengths. Its cross‐sectional design enabled the identification of associations between specific types of MUPI and parenting outcomes. Including parents of children aged 6–19 years also broadened the scope beyond the typical focus on early childhood.

However, several limitations should be acknowledged. First, the cross‐sectional design prevents causal inference; longitudinal studies are needed to examine how media use influences parenting over time. Second, the sample, primarily recruited online, may not be representative, likely overrepresenting digitally literate, middle‐aged and highly educated parents, which could have inflated the observed associations. Future research should aim for more diverse and representative samples through both online and offline recruitment. Third, reliance on self‐report measures introduces risks of recall bias and social desirability. Using additional sources, such as child reports, observational methods, or digital tracking, could enhance validity. Fourth, although the MUPI instrument demonstrated excellent content validity, its internal consistency was modest, likely due to floor effects in certain rarely used media modalities. Future research should further assess its reliability through test–retest methods and explore analytic approaches that better account for heterogeneous media‐use patterns. Lastly, as AI tools increasingly offer personalised parenting support, future research should explore their role in fostering positive parenting behaviours.

## 5 Implication for Practice, Application, Theory and Polity

As digital media platforms become increasingly central to parenting education, health professionals may play an important role in guiding parents toward evidence‐based, expert‐led resources, especially text‐based materials, structured training and professionally moderated social media platforms. Short‐form videos may be best viewed as supplementary tools rather than primary sources of parenting guidance. Digital content creators could improve short‐form materials by producing series‐based, chapter‐specific content that supports structured learning and by embedding links to reliable resources. Government and public health agencies may benefit from prioritising user‐centred design and regular content updates to enhance the accessibility and relevance of digital parenting platforms.

## Author Contributions

**Pornnapasorn Nguansiri:** conceptualisation, methodology, data curation, formal analysis, writing – original draft. **Komsan Kiatrungrit:** conceptualisation, methodology, supervision, writing – original draft, writing – review and editing. **Sirichai Hongsanguansri:** conceptualisation, methodology, writing – review and editing. **Nitchawan Jongrakthanakij:** conceptualisation, methodology, writing – review and editing. **Wanlop Atsariyasing:** conceptualisation, methodology, writing – review and editing. **Vilawan Chirdkiatgumchai:** conceptualisation, methodology, writing – review a editing. **Chosita Pavasuthipaisit:** conceptualisation, methodology, writing – review and editing.

## Funding

The authors received no specific funding for this work.

## Ethics Statement

The authors affirm that all procedures contributing to this work comply with the ethical standards of relevant institutional and national guidelines on human experimentation. Informed consent was obtained from all individual participants included in the study. Ethical approval for the study was granted by the Institutional Review Board, approval number: MURA2024/380. The study ensured the ethical treatment of participants, respect for their rights and protection of personal data. No identifying information of individual participants is included in the reported results or accessible through publicly available data.

## Consent

All participants provided written informed consent prior to their enrolment in the study. The consent process included a clear explanation of the study's objectives, procedures, potential risks and benefits, confidentiality safeguards and the voluntary nature of participation. No identifying personal data are included in the manuscript, and all responses were anonymised prior to analysis.

## Conflicts of Interest

The authors declare no conflicts of interest.

## Supporting information


**Data S1:** Self‐report media use for parenting information (English).
**Data S2:** Child and Adolescent parenting Knowledge Evaluation (CAKE) (English).
**Table S1:** Preferred sources of parenting information.
**Table S2:** Differences between media use for parenting information among parents of school age children and parents of teenagers.
**Table S3:** Linear regression analysis of media use for parenting information (MUPI) on parenting knowledge, attitudes and practices among parents of school‐age children and parents of teenagers.

## Data Availability

The data that support the findings of this study are available from the corresponding author upon reasonable request. Due to ethical considerations and the inclusion of sensitive participant information, the dataset is not publicly shared to protect the confidentiality and privacy of respondents.

## References

[cch70233-bib-0001] Anisa, N. R. I. , R. T. Wulandar , and T. Iriyanto . 2023. “The Effectiveness of Parenting Content on Tik Tok Social Media on Parenting Knowledge.” In Proceedings of the 1st International Conference on Early Childhood Education in Multiperspective, edited by F. F. M. Roqib , S. Sunhaji , and H. Kurniawan , 23. EAI.

[cch70233-bib-0002] Archaphet, N. , Pharksuwan, M. , & Damrongkiattisak, P. (2021). Sharenting: the Parents' Overlooked Children Rights Issues. RMUTL Journal of Business Administration and Liberal Arts, 9(2), 131–146. Retrieved from https://so05.tci‐thaijo.org/index.php/balajhss/article/view/253753.

[cch70233-bib-0003] Areemit, R. , P. Lumbiganon , C. Suphakunpinyo , A. Jetsrisuparb , S. Sutra , and K. Sripanidkulchai . 2020. “A Mobile App, KhunLook, to Support Thai Parents and Caregivers With Child Health Supervision: Development, Validation, and Acceptability Study.” JMIR mHealth and uHealth 8, no. 10: e15116. 10.2196/15116. 33124989 PMC7665943

[cch70233-bib-0004] Areemit, R. , S. Saengnipanthkul , S. Sutra , et al. 2023. “Effectiveness of a Mobile App (KhunLook) Versus the Maternal and Child Health Handbook on Thai Parents' Health Literacy, Accuracy of Health Assessments, and Convenience of Use: Randomized Controlled Trial.” Journal of Medical Internet Research 25: e43196. 10.2196/43196. 37159258 PMC10206628

[cch70233-bib-0005] Baku, E. A. , I. Agbemafle , and R. M. K. Adanu . 2017. “Effects of Parents Training on Parents' Knowledge and Attitudes About Adolescent Sexuality in Accra Metropolis, Ghana.” Reproductive Health 14, no. 1: 101. 10.1186/s12978-017-0363-9. 28836984 PMC5571628

[cch70233-bib-0006] C. S. Mott Children's Hospital University of Michigan Health . 2023. “Sharing on Parenting: Getting Advice Through Social Media.” Retrieved from Ann Arbor, MI: https://www.mottchildren.org/news/releases/sharing‐on‐parenting‐getting‐advice‐through‐social‐media.

[cch70233-bib-0007] Chen, Y. , Mingming, L. , Fu, G. , & and Wang, X. (2023). The Effect of Short‐Form Video Addiction on Users' Attention. Behaviour & Information Technology, 42(16), 2893–2910. 10.1080/0144929X.2022.2151512.

[cch70233-bib-0008] Chua, J. Y. X. , and S. Shorey . 2022. “Effectiveness of Mobile Application‐Based Perinatal Interventions in Improving Parenting Outcomes: a Systematic Review.” Midwifery 114: 103457. 10.1016/j.midw.2022.103457. 35985142 PMC9364944

[cch70233-bib-0009] David, O. A. , I. A. Iuga , and I. S. Miron . 2024. “Parenting: There Is an App for That. A Systematic Review of Parenting Interventions Apps.” Children and Youth Services Review 156: 107385. 10.1016/j.childyouth.2023.107385.

[cch70233-bib-0010] Duong, H. T. , L. T. Van Nguyen , H. T. Vu , and A. T. Trinh . 2020. “Association Between Online Social Influence and Corporal Punishment: an Experimental Study.” Child and Adolescent Social Work Journal 37, no. 2: 163–177. 10.1007/s10560-019-00632-9.

[cch70233-bib-0011] El Sherif, R. , P. Pluye , V. Paquet , F. Ibekwe , and R. Grad . 2023. “How People Use Web‐Based Parenting Information to Support Others in Their Social Circle: Qualitative Descriptive Study.” JMIR Pediatrics and Parenting 6: e40043. 10.2196/40043. 37115603 PMC10182472

[cch70233-bib-0012] Fierloos, I. N. , Windhorst, D. A. , Fang, Y. , Mao, Y. , Crone, M. R. , Hosman, C. M. H. , . . . Raat, H. (2022). Factors Associated With Media Use for Parenting Information: a Cross‐Sectional Study Among Parents of Children Aged 0–8 Years. Nursing Open, 9(1), 446–457. 10.1002/nop2.1084. 34672428 PMC8685885

[cch70233-bib-0013] Frey, E. , C. Bonfiglioli , M. Brunner , and J. Frawley . 2022. “Parents' Use of Social Media as a Health Information Source for Their Children: a Scoping Review.” Academic Pediatrics 22, no. 4: 526–539. 10.1016/j.acap.2021.12.006. 34906742

[cch70233-bib-0014] Frick, P. J. 1991. “Alabama Parenting Questionnaire (APQ) (Publication no. 10.1037/t58031‐000).” Retrieved July 30, 2024, from APA PsycTests https://psycnet.apa.org/doiLanding?doi=10.1037%2Ft58031‐000.

[cch70233-bib-0015] Gibaud‐Wallston, J. , and L. P. Wandersman 1978. “Development and Utility of the Parenting Sense of Competence Scale.” Paper Presented at the The Annual Meeting of the American Psychological Association, Toronto.

[cch70233-bib-0016] Harris, L. E. , and J. A. Jacobs . 2023. “Emerging Ideas. Digital Parenting Advice: Online Guidance Regarding Children's use of the Internet and Social Media.” Family Relations 72, no. 5: 2551–2568. 10.1111/fare.12813.

[cch70233-bib-0017] Kessara, K. , S. Saenwa , and P. Nilsook . 2024. “An Analysis Relationship of the Usability Factors of Government Ministry Websites in Thailand.” Journal of Humanities and Social Sciences Thonburi University 18, no. 3: 52–61. https://so03.tci‐thaijo.org/index.php/trujournal/article/view/274753.

[cch70233-bib-0018] Khamon, A. , C. Charnsil , M. Srisurapanont , and C. Suradom . 2019. “Development and Validation of the Thai Version of the Alabama Parenting Questionnaire (APQ).” Journal of Mental Health of Thailand 27, no. 2: 107–120. https://he01.tci‐thaijo.org/index.php/jmht/article/view/204133.

[cch70233-bib-0019] Kostyrka‐Allchorne, K. , C. Ballard , S. Byford , et al. 2022. “Online Parent Training for The Initial Management of ADHD Referrals (OPTIMA): the Protocol for a Randomised Controlled Trial of a Digital Parenting Intervention Implemented to Support Parents and Children on a Treatment Waitlist.” Trials 23, no. 1: 1003. 10.1186/s13063-022-06952-z. 36510236 PMC9744042

[cch70233-bib-0020] Kubb, C. , and H. M. Foran . 2020. “Online Health Information Seeking by Parents for Their Children: Systematic Review and Agenda for Further Research.” Journal of Medical Internet Research 22, no. 8: e19985. 10.2196/19985. 32840484 PMC7479585

[cch70233-bib-0021] Liu, H. X. , K. T. Lee , and S. Bai . 2023. “Exploring Motivations for TikTok Usage and Impact Factors of TikTokers' Continuance Intention.” Ingénierie des Systèmes D'information 28, no. 2: 389–400. 10.18280/isi.280214.

[cch70233-bib-0022] Mause, L. , J. Hoffmann , A. Reimer , et al. 2022. “Trust in Medical Professionals and Its Influence on the Stress Experience of Parents of Premature Infants.” Acta Paediatrica 111, no. 3: 527–535. 10.1111/apa.16187. 34779058

[cch70233-bib-0023] Mertens, E. , G. Ye , E. Beuckels , and L. Hudders . 2024. “Parenting Information on Social Media: Systematic Literature Review.” JMIR Pediatr Parent 7: e55372. 10.2196/55372. 39442173 PMC11541157

[cch70233-bib-0024] Moungchinda P. 2022. “The Role of TikTok Content Creators on Decision Made for Children's Health and Wellbeing From Thai Mothers' Point of View: a Case Study.” (Bachelor Text). Thammasat University, https://digital.library.tu.ac.th/tu_dc/frontend/Info/item/dc:308229.

[cch70233-bib-0025] National Academies of Sciences, E., Medicine, Division of, B., Social, S., Education, Board on Children, Y., … Committee on Supporting the Parents of Young, C . 2016. In Parenting Matters: Supporting Parents of Children Ages 0–8, edited by H. Breiner , M. Ford , and V. L. Gadsden . National Academies Press (US) Copyright 2016 by the National Academy of Sciences. 27997088

[cch70233-bib-0026] Onishi, R. 2024. “Parental Information‐Use Strategies in a Digital Parenting Environment and Their Associations With Parental Social Support and Self‐Efficacy: Cross‐Sectional Study.” JMIR Pediatr Parent 7: e58757. 10.2196/58757. 39700496 PMC11695971

[cch70233-bib-0027] Puridej, E. 2025. “Do Thais Enjoy Social Media Just for Fun? Evolving Social Media Trends and Thai Digital Citizenship, Internet.” *The Nation* Retrieved from https://www.nationthailand.com/blogs/news/general/40045288.

[cch70233-bib-0028] Reyes, D. R. G. , R. M. Jocson , L. Peña Alampay , B. Landoy Mamauag , J. C. Reyes , and J. M. Lachman . 2024. “Evaluation of a Brief Online Parenting Training for Community Service Providers in the Philippines.” Children and Youth Services Review 161: 107664. 10.1016/j.childyouth.2024.107664.

[cch70233-bib-0029] Rugrien, P. 2022. “ Social Media Trend 2023: Short‐Form vs. Long‐Form Video .” (Undergraduate Bachelor's Thesis). Thammasat University. https://digital.library.tu.ac.th/tu_dc/frontend/Info/item/dc:308246.

[cch70233-bib-0030] Ryan, R. , C. O'Farrelly , and P. Ramchandani . 2017. “Parenting and Child Mental Health.” London J Prim Care (Abingdon) 9, no. 6: 86–94. 10.1080/17571472.2017.1361630. PMC5694794 29181091

[cch70233-bib-0031] Sanders, M. R. , and K. M. T. Turner . 2018. “The Importance of Parenting in Influencing the Lives of Children.” In Handbook of Parenting and Child Development Across the Lifespan, edited by M. R. Sanders and A. Morawska , 3–26. Cham: Springer International Publishing.

[cch70233-bib-0032] Settachan, D. 2021. “Factors That Influence People to Use TikTok in Thailand.” (Master). Mahidol University, Bangkok, Thailand. https://archive.cm.mahidol.ac.th/bitstream/123456789/4175/1/TP%20MM.014%202021.pdf.

[cch70233-bib-0033] Setyastuti, Y. , J. R. Suminar , P. Hadisiw , and F. Zubair . 2019. “Millennial Moms: Social Media as the Preferred Source of Information About Parenting in Indonesia.” Library Philosophy and Practice: 2558. https://digitalcommons.unl.edu/libphilprac/2558/.

[cch70233-bib-0034] Strehlke, E. , R. Bromme , and J. Kärtner . 2025. “Whom to Ask? Whom to Trust? Parents' Preferences for Sources of Advice on Social‐Emotional Parenting Issues.” Counselling Psychology Quarterly 38, no. 1: 1–20. 10.1080/09515070.2024.2331441.

[cch70233-bib-0035] Sullivan, J. A. , B. J. Zvara , S. A. Keim , R. Andridge , and S. E. Anderson . 2021. “Knowledge of Infant Development and Parent Well‐Being: Cross‐Sectional Analysis of Toddlers.” Journal of Developmental and Behavioral Pediatrics 42, no. 6: 442–449. 10.1097/dbp.0000000000000918. 34397572 PMC8371675

[cch70233-bib-0036] Suwansujarid, T. , P. Vatanasomboon , N. Gaylord , and P. Lapvongwatana . 2013. “Validation of the Parenting Sense of Competence Scale in Fathers: Thai Version.” Southeast Asian Journal of Tropical Medicine and Public Health 44, no. 5: 916–926. 24437327

[cch70233-bib-0037] Taylor, C. A. , R. Al‐Hiyari , S. J. Lee , A. Priebe , L. W. Guerrero , and A. Bales . 2016. “Beliefs and Ideologies Linked With Approval of Corporal Punishment: a Content Analysis of Online Comments.” Health Education Research 31, no. 4: 563–575. 10.1093/her/cyw029. 27312115 PMC4945859

[cch70233-bib-0038] Taylor, C. A. , L. Hamvas , J. Rice , D. L. Newman , and W. DeJong . 2011. “Perceived Social Norms, Expectations, and Attitudes Toward Corporal Punishment Among an Urban Community Sample of Parents.” Journal of Urban Health 88, no. 2: 254–269. 10.1007/s11524-011-9548-7. 21336503 PMC3079037

[cch70233-bib-0039] Vale‐Dias, M. , and L. Nobre‐Lima . 2018. “Parents Knowledge About the Development of Children Aged 2 to 6 Years Old.” International Journal of Developmental and Educational Psychology. Revista INFAD de Psicología 4: 149. 10.17060/ijodaep.2018.n1.v4.1284.

[cch70233-bib-0040] Wang, S. , and A. Inkuer . 2024. “The Role of Aesthetic Design in Enhancing User Engagement and Retention on Digital Exhibition Platform.” Journal of Roi Kaensarn Academi 9, no. 12: 853–865. https://so02.tci‐thaijo.org/index.php/JRKSA/article/view/271234.

[cch70233-bib-0041] Xiao, L. , X. Li , and Y. Zhang . 2023. “Exploring the Factors Influencing Consumer Engagement Behavior Regarding Short‐Form Video Advertising: a Big Data Perspective.” Journal of Retailing and Consumer Services 70: 103170. 10.1016/j.jretconser.2022.103170.

[cch70233-bib-0042] Yamanaka, T. , K. Yuruki , Y. Sanabe , M. Yasutake , and M. Inoue . 2023. “Assessing the Effectiveness of Real‐Time Online Parent Training for Parents of Children With Diverse Neurodevelopmental Disorders Residing in the Community.” Yonago Acta Medica 66, no. 4: 448–458. 10.33160/yam.2023.11.010. 38028270 PMC10674063

[cch70233-bib-0043] Yamashita, A. , A. Isumi , and T. Fujiwara . 2022. “Online Peer Support and Well‐Being of Mothers and Children: Systematic Scoping Review.” Journal of Epidemiology 32, no. 2: 61–68. 10.2188/jea.JE20200079. 33132282 PMC8761562

[cch70233-bib-0044] Yan, T. , C. Su , W. Xue , Y. Hu , and H. Zhou . 2024. “Mobile Phone Short Video Use Negatively Impacts Attention Functions: an EEG Study.” Frontiers in Human Neuroscience 18: 1383913. 10.3389/fnhum.2024.1383913. 38993329 PMC11236742

[cch70233-bib-0045] Zhang, C. , H. Zheng , and Q. Wang . 2022. “Driving Factors and Moderating Effects Behind Citizen Engagement With Mobile Short‐Form Videos.” IEEE Access 10: 40999–41009. 10.1109/ACCESS.2022.3167687.

