# Supplementary material for: The Association Between Media Use for Parenting Information (MUPI) and Parenting Knowledge, Attitudes and Practices Among Parents of Children Aged 6–19 Years
Source: Child Care Health Dev. 2026 Jan 29;52(2):e70233. doi: 10.1111/cch.70233 (PMC12856387; doi:10.1111/cch.70233)
Supplement: Supplementary file 1 — Data S1: Self‐report media use for parenting information (English). Data S2: Child and Adolescent parenting Knowledge Evaluation (CAKE) (English). Table S1: Preferred sources of parenting information. Table S2: Differences between media use for parenting information among parents of school age children and parents of teenagers. Table S3: Linear regression analysis of media use for parenting information (MUPI) on parenting knowledge, attitudes and practices among parents of school‐age children and parents of teenagers. [file CCH-52-e70233-s001.docx]

**The Association Between Media Use for Parenting Information (MUPI) and Parenting Knowledge, Attitudes and Practices Among Parents of Children Aged 6-19 Years**

Supplemental Materials

Table of Contents

| 1. Research Instruments |  |
| --- | --- |
| 1.1 Questionnaire S1: Self-report Media Use for Parenting Information (Thai) | 3 |
| 1.2 Questionnaire S1: Self-report Media Use for Parenting Information (English) | 4 |
| 1.3 Questionnaire S2: Child and Adolescent parenting Knowledge Evaluation (CAKE)   (Thai) | 5 |
| 1.4 Questionnaire S2: Child and Adolescent parenting Knowledge Evaluation (CAKE)  (English) | 10 |
| 2. Supplementary Table |  |
| 2.1 Table S1: Preferred sources of parenting information  2.2 Table S2: Differences between media use for parenting information  among parents of school age children and parents of teenagers  2.3 Table S3: Linear regression analysis of media use for parenting information (MUPI)   on parenting knowledge, attitudes and practices among parents of school-age   children and parents of teenagers | 14  15  16 |

**Questionnaire S1: Self-report Media Use for Parenting Information (Thai)**

แบบสอบถามการใช้สื่อเพื่อการเลี้ยงดูบุตร

แบบสอบถามนี้ มีคำถามทั้งหมด 9 ข้อโปรดอ่านคำถามต่อไปนี้อย่างละเอียด และเลือกตอบข้อที่ท่านคิดว่าเหมาะสมมากที่สุด เพียง 1 ข้อ

**ส่วนที่ 1) การเลือกใช้สื่อ (ข้อ 1)**

______________________________________________________________________________

1.เมื่อคิดถึงข้อมูลการเลี้ยงดูบุตรที่เคยได้รับมา ท่านชื่นชอบการรับฟังข้อมูลจากช่องทางใดมากที่สุด

สื่อที่ผลิตโดยหน่วยงานรัฐบาล

สื่อที่ผลิตโดยโรงพยาบาลรัฐ หรือ เอกชน

สื่อที่ผลิตโดยบุคลากรทางการแพทย์

สื่อที่ผลิตโดยบุคคลทั่วไปเช่น ผู้ปกครอง ดารา

**ส่วนที่ 2) กิจกรรมการใช้สื่อเพื่อการเลี้ยงดูบุตร (ข้อ 2-9)**

คำชี้แจง: ใน 3 เดือนที่ผ่านมา ท่านหาข้อมูลการเลี้ยงดูบุตรผ่านกิจกรรมแต่ละอย่าง บ่อยเพียงใด

2.การอ่านสิ่งพิมพ์ (เช่น หนังสือ ตำรา คู่มือ)

ไม่ได้ใช้ 1-4 วัน/เดือน 5-7 วัน/ เดือน 1-4 วัน/สัปดาห์ 5-7 วัน/ สัปดาห์

3.การอ่านสื่อออนไลน์ (เช่น Pantip, Facebook หรือ Social Media และเว็ปไซต์อื่นๆ)

ไม่ได้ใช้ 1-4 วัน/เดือน 5-7 วัน/ เดือน 1-4 วัน/สัปดาห์ 5-7 วัน/ สัปดาห์

4.การดูวิดีโอคลิปยาว (เช่น YouTube, NetPama, Facebook Live)

ไม่ได้ใช้ 1-4 วัน/เดือน 5-7 วัน/ เดือน 1-4 วัน/สัปดาห์ 5-7 วัน/ สัปดาห์

5.การดูวิดีโอคลิปสั้น (เช่น TikTok, Reels)

ไม่ได้ใช้ 1-4 วัน/เดือน 5-7 วัน/ เดือน 1-4 วัน/สัปดาห์ 5-7 วัน/ สัปดาห์

6.การฟัง (เช่น คลิปเสียง รายการวิทยุ พอดแคสต์ )

ไม่ได้ใช้ 1-4 วัน/เดือน 5-7 วัน/ เดือน 1-4 วัน/สัปดาห์ 5-7 วัน/ สัปดาห์

7.การตั้งคำถามผ่านสื่อสังคมออนไลน์ (Social Media) เช่นโพสต์ คอมเมนต์ ส่งข้อความ ตั้งกระทู้

ไม่ได้ใช้ 1-4 วัน/เดือน 5-7 วัน/ เดือน 1-4 วัน/สัปดาห์ 5-7 วัน/ สัปดาห์

8.การเข้าคอร์สอบรมออนไลน์ (เช่น ผ่าน Zoom, Webex, Google Meet)

ไม่ได้ใช้ 1-4 วัน/เดือน 5-7 วัน/ เดือน 1-4 วัน/สัปดาห์ 5-7 วัน/ สัปดาห์

9.การใช้แอพพลิเคชั่นการเลี้ยงดูบุตร (เช่น Khunlook, Parents, The Happy Child Parenting App)

ไม่ได้ใช้ 1-4 วัน/เดือน 5-7 วัน/ เดือน 1-4 วัน/สัปดาห์ 5-7 วัน/ สัปดาห์

**Questionnaire S1: Self-report Media Use for Parenting Information (English)**

**Self-report Media Use for Parenting Information**

This questionnaire contains 9 items. Please read each question carefully and select the single response that best represents your answer.

**Section 1) Media Selection (Items 1-2)**

______________________________________________________________________________

1. When thinking about the parenting information you have previously received, through which channel do you most prefer to receive such information? *(Select one.)*
   □ Media produced by government agencies
   □ Media produced by public or private hospitals
   □ Media produced by healthcare professionals (e.g., physicians, nurses, psychologists)
   □ Media produced by the general public (e.g., parents, celebrities)

**Section 2) Media-Use Activities for Parenting (Items 2–9)**
*Instruction:* In the past 3 months, how often did you seek parenting information through each of the following activities?

1. Reading print materials (e.g., books, textbooks, manuals)
   □ Never □ 1–4 days/month □ 5–7 days/month □ 1–4 days/week □ 5–7 days/week
2. Reading online media (e.g., via Facebook, Pantip or other social media and websites)
   □ Never □ 1–4 days/month □ 5–7 days/month □ 1–4 days/week □ 5–7 days/week
3. Watching long-form video clips (e.g., via YouTube, NetPama, Facebook Live)
   □ Never □ 1–4 days/month □ 5–7 days/month □ 1–4 days/week □ 5–7 days/week
4. Watching short-form video clips (e.g., via TikTok, Reels)
   □ Never □ 1–4 days/month □ 5–7 days/month □ 1–4 days/week □ 5–7 days/week
5. Listening to audio media (e.g., audio clips, radio programs, podcasts)
   □ Never □ 1–4 days/month □ 5–7 days/month □ 1–4 days/week □ 5–7 days/week
6. Asking questions via social media (e.g., posting, commenting, messaging, initiating a discussion)
   □ Never □ 1–4 days/month □ 5–7 days/month □ 1–4 days/week □ 5–7 days/week
7. Attending online training courses (e.g., via Zoom, Webex, Google Meet)
   □ Never □ 1–4 days/month □ 5–7 days/month □ 1–4 days/week □ 5–7 days/week
8. Using parenting applications (e.g., Khunlook, Parents, The Happy Child Parenting App)
   □ Never □ 1–4 days/month □ 5–7 days/month □ 1–4 days/week □ 5–7 days/week

**Questionnaire S2: Child and Adolescent parenting Knowledge Evaluation (CAKE) (Thai)**

แบบประเมินความรู้ผู้ปกครองในการเลี้ยงดูเด็กและวัยรุ่น  มีคำถามทั้งหมด 20  ข้อ โปรดอ่านคำถามต่อไปนี้อย่างละเอียดและเลือกตอบข้อที่ท่านคิดว่าเหมาะสมมากที่สุด **เพียง 1 ข้อ**

**1**. เมื่อบุตรหลานเข้าสู่วัยเรียน-วัยรุ่น ผู้ปกครองควรให้ความสำคัญและสนับสนุนเรื่องใดมากที่สุด

A. การมีผลการเรียนดีและกิจกรรมเด่น

B. การดูแลบุคลิกภาพและรูปร่างหน้าตา

C. การแบ่งเบาภาระสมาชิกในครอบครัว

D. การทำตามความคาดหวังของครอบครัว

E. การค้นหาตัวเองผ่านกิจกรรมหลากหลาย

2. ข้อใดเป็นจุดประสงค์หลักของการพูดคุยเรื่องเพศกับบุตรหลาน

A. เพื่อให้เด็กไม่มีความลับกับผู้ปกครอง

B. เพื่อควบคุมพฤติกรรมทางเพศของเด็ก

C. เพื่อป้องกันการมีเพศสัมพันธ์ในวัยเรียน

D. เพื่อให้เด็กระมัดระวังตัวจากเพศตรงข้าม

E. เพื่อให้ข้อมูลที่ถูกต้องและเหมาะสมกับวัย

3. บุตรหลานวัย 8 ปีเป็นเด็กขี้กังวล อยากลงแข่งกีฬาแต่กลัวความล้มเหลว ผู้ปกครองควรปฏิบัติอย่างไรมากที่สุด

A. ชวนพูดคุยเรื่องที่เด็กกังวล

B. ให้ทำกิจกรรมที่เด็กมั่นใจแทน

C. ปล่อยให้เด็กตัดสินใจด้วยตนเอง

D. บอกว่าจะให้รางวัลถ้าเด็กยอมลงแข่ง

E. ยืนยันให้เด็กลงแข่งเพื่อเอาชนะความกลัว

4. ผู้ปกครองควรให้อิสระวัยรุ่นในการตัดสินใจอย่างไร

A. ให้วัยรุ่นเป็นคนตัดสินใจปัญหาครอบครัว

B. กำหนดขอบเขตเรื่องที่วัยรุ่นตัดสินใจเองได้

C. ให้อิสระวัยรุ่นตัดสินใจได้เฉพาะเรื่องส่วนตัว

D. ให้วัยรุ่นมาปรึกษาก่อนที่จะตัดสินใจทุกเรื่อง

E. ผู้ปกครองตัดสินใจเรื่องเรียนและกิจกรรมให้วัยรุ่น

5. บุตรหลานวัย 15 ปี ท่านพบภาพและคลิปวิดีโอโป๊เปลือยของบุคคลที่เป็นเพศเดียวกันจึงสงสัยว่าเขาจะรักหรือชอบเพศเดียวกันหรือไม่ ท่านควรปฏิบัติอย่างไรมากที่สุด

A. รอให้บุตรหลานมาพูดคุยเรื่องเพศด้วยตัวเอง

B. พาไปปรึกษาแพทย์เรื่องการเบี่ยงเบนทางเพศ

C. ขอให้บุตรหลานลบภาพและคลิปวิดีโอดังกล่าว

D. เรียกบุตรหลานมาตำหนิเกี่ยวกับรสนิยมทางเพศ

E. หาโอกาสพูดคุยเรื่องความสนใจทางเพศของบุตรหลาน

6. เมื่อทราบว่าบุตรหลานวัย 9 ปี ถูกกลั่นแกล้ง (เช่นล้อเลียน, ผลัก หรือ ขู่เอาทรัพย์สิน) ที่โรงเรียน ท่านควรปฎิบัติอย่างไรเป็นลำดับแรก

A. ให้เด็กเอาตัวรอดด้วยตนเอง

B. สอนให้ลูกเพิกเฉยการกลั่นแกล้ง

C. สอนให้อดทนเมื่อเพื่อนมาล้อเลียน

D. ไปพบครูเพื่อจัดการเด็กที่แกล้งบุตรหลาน

E. ชวนคุยวิธีป้องกันและรับมือกับการกลั่นแกล้ง

7. บุตรหลานวัย 10 ปี มีรูปร่างอ้วนจนเคลื่อนไหวไม่สะดวก ผู้ปกครองควรปฏิบัติอย่างไรมากที่สุด

A. หมั่นพูดข้อเสียของน้ำหนักตัวเด็ก

B. งดขนมหวานและพาไปออกกำลังกาย

C. ให้เด็กเรียนรู้ผลเสียต่อสุขภาพด้วยตนเอง

D. พาเด็กไปพบแพทย์เพื่อปรึกษาเรื่องน้ำหนัก

E. ให้เด็กมีส่วนร่วมในการปรับอาหารและกิจกรรม

8. ช่วง 2 สัปดาห์ที่ผ่านมา บุตรหลานวัย 13 ปี เก็บตัวในห้อง เล่นเกมมากกว่าปกติ มักหงุดหงิดง่ายและโมโหรุนแรง ไม่มีสมาธิในการอ่านหนังสือจนผลการเรียนตก ผู้ปกครองควรปฏิบัติอย่างไรมากที่สุด

A. ตักเตือนให้เด็กปรับปรุงตัว

B. จัดตารางเวลาเล่นเกมและเข้านอน

C. ลดเวลาเล่นเกมจนกว่าผลการเรียนจะดีขึ้น

D. คอยเปิดประตูห้องนอนเพื่อดูว่าเด็กทำอะไร

E. สอบถามเรื่องอารมณ์และพฤติกรรมที่เปลี่ยนไป

9. เมื่อท่านพบแผลเป็นที่สงสัยว่าเกิดจากการกรีดแขนของบุตรหลานวัยรุ่น ท่านควรปฏิบัติอย่างไรเป็นอันดับแรก

A. สอบถามจนกว่าเด็กจะยอมเล่า

B. ดูแลความปลอดภัยไม่ให้คลาดสายตา

C. บอกความรู้สึกว่าท่านเสียใจกับสิ่งที่เขาทำ

D. สอบถามเรื่องราว ให้เวลาเด็กพร้อมที่จะเล่า

E. สอนว่าการทำร้ายตนเองเป็นสิ่งที่ไม่เหมาะสม

10. เมื่อทราบว่าบุตรหลานวัย 15 ปี มักไม่เข้าเรียน สอบตกหลายวิชา และยกพวกตีกับเพื่อนในโรงเรียน ท่านควรปฏิบัติอย่างไรมากที่สุด

A. พูดคุยเรื่องผลเสียของพฤติกรรม

B. ให้เรียนรู้ผลที่จะตามมาด้วยตนเอง

C. กำหนดบทลงโทษต่อการใช้ความรุนแรง

D. ชวนคุยเรื่องทัศนคติต่อเพื่อนและพฤติกรรม

E. ขอให้บุตรหลานเลิกคบกับเพื่อนกลุ่มปัจจุบัน

11.ในการฝึกวินัย ผู้ปกครองควรให้ความสำคัญกับเรื่องใดมากที่สุด

A. การลงโทษรุนแรงเพื่อให้เด็กสำนึกผิด

B. การจัดการปัญหาพฤติกรรมให้หมดไป

C. การให้เด็กรู้จักเก็บอารมณ์และความรู้สึก

D. การให้เด็กเชื่อฟังคำสั่งสอนของผู้ปกครอง

E. การส่งเสริมทักษะการควบคุมตนเองของเด็ก

12.  เด็กวัย 8 ปี เมื่อถึงเวลาปิดคอมพิวเตอร์ มักขอต่อเวลา ผู้ปกครองควรปฏิบัติอย่างไรมากที่สุด

A. ปิดคอมพิวเตอร์ทันทีเมื่อหมดเวลา

B. งดพาไปเที่ยวในวันหยุดเสาร์-อาทิตย์

C. เก็บคอมพิวเตอร์และไม่อนุญาตให้ใช้อีก

D. ไม่อนุญาตให้เล่นและหากิจกรรมอื่นให้ทำแทน

E. ตกลงกันก่อนว่าเด็กต้องรับผิดชอบอย่างไรถ้าใช้เกินเวลา

13. บุตรหลานวัย 11 ปี มักเถียง โวยวาย และพูดคำหยาบเมื่อถูกขัดใจ ขณะที่เด็กมีพฤติกรรมดังกล่าว ผู้ปกครองควรปฏิบัติอย่างไรมากที่สุด

A. บอกให้เด็กหยุดพฤติกรรมทันที

B. อธิบายจนกว่าเด็กจะเข้าใจเหตุผล

C. กล่าวตักเตือนว่าพฤติกรรมไม่เหมาะสม

D. เตือนทันทีว่าไม่ควรพูดแบบนี้กับผู้ใหญ่

E. เตือนให้ลูกไปสงบอารมณ์ก่อนค่อยมาคุยกัน

14. บุตรหลานวัย 12 ปี มักตื่นสาย ใช้เวลานานในการอาบน้ำ แต่งตัว ทานข้าวเช้า จนไปโรงเรียนสายบ่อยครั้ง ผู้ปกครองควรปฏิบัติอย่างไรมากที่สุด

A. ปลุกก่อนเวลาเดิม 15-30 นาที

B. ลงโทษให้หนักขึ้นในครั้งต่อๆไป

C. สร้างกติกาและบทลงโทษร่วมกันใหม่

D. คอยเร่งให้อาบน้ำ แต่งตัว ทานข้าวเช้า

E. ตกลงเวลาออกจากบ้านและรางวัลที่ได้รับเมื่อปฏิบัติได้

15.บุตรหลานวัย 17 ปี ขอไปร่วมงานเลี้ยงกลางคืนที่บ้านเพื่อนและนอนค้างกับกลุ่มเพื่อน เมื่อท่านกังวลเรื่องการดื่มแอลกอฮอลล์ ควรปฏิบัติอย่างไรมากที่สุด

A. ให้ไปงานโดยขอไม่ให้ดื่มแอลกอฮอล์

B. บอกความกังวลและไม่อนุญาตให้ไปงานเลี้ยง

C. บอกว่าจะลงโทษหากทราบว่าดื่มแอลกอฮอล์

D. ชวนคุยเรื่องความเสี่ยงที่อาจเกิดขึ้นจากการดื่ม

E. ชวนทำกิจกรรมอื่นที่ผู้ปกครองคิดว่าเหมาะสมแทน

16. บุตรหลานวัย 13 ปี โกรธน้องสาวที่มารบกวนขณะเล่นเกม จึงตีน้องเด็กสารภาพว่าทำตามเกม ผู้ปกครองควรปฏิบัติอย่างไรมากที่สุด

A. ลงโทษพี่ที่ทำรุนแรงกับน้อง

B. กำหนดเกมที่บุตรหลานสามารถเล่นได้

C. งดการเล่นเกมและให้ทำกิจกรรมอื่นแทน

D. ชวนคุยเรื่องเกมว่าอะไรควรหรือไม่ควรทำ

E. ให้ดูสื่อเฉพาะเมื่ออยู่ในสายตาของผู้ปกครอง

17. ข้อใดเป็นการดูแลการใช้สื่อของบุตรหลานอายุ 7 ปี ที่เหมาะสมที่สุด

A. กำหนดเวลาการใช้สื่อต่อวัน

B. ให้ดูรายการเพื่อการศึกษาเท่านั้น

C. ให้ดูสื่อบันเทิงได้เฉพาะในวันหยุด

D. ให้เด็กเลือกรายการที่จะดูได้อย่างอิสระ

E. ให้เวลาดูทีวีเพิ่มถ้าเด็กทำการบ้านเสร็จเร็ว

18.ผู้ปกครองควรเป็นแบบอย่างในการใช้สื่อให้กับบุตรหลานอย่างไร

A. ดูข่าวลำพังในห้องนอน

B. ดูทีวีขณะรับประทานอาหารร่วมกัน

C. เปลี่ยนช่องทันทีเมื่อพบความรุนแรงในสื่อ

D. วิจารณ์พฤติกรรมไม่เหมาะสมที่พบเห็นในทีวี

E. แบ่งเวลาดูทีวีและทำกิจกรรมกับลูกอย่างเหมาะสม

19. ในการดูแลใช้สื่อสังคมออนไลน์ (social media) ของเด็กวัยเรียน ผู้ปกครองควรปฏิบัติอย่างไรมากที่สุด

A. ให้ใช้สื่อสังคมออนไลน์ในสายตาผู้ปกครอง

B. ให้ใช้บัญชีสื่อสังคมออนไลน์เดียวกับผู้ปกครอง

C. ตำหนิเมื่อพบว่าบุตรหลานดูเนื้อหาที่ไม่เหมาะสม

D. ไม่อนุญาตให้ใช้สื่อสังคมออนไลน์ก่อนอายุ 12 ปี

E. ชวนคุยสิ่งที่ควรและไม่ควรทำบนสื่อสังคมออนไลน์

20. ในการดูแลใช้สื่อสังคมออนไลน์ (social media) ของวัยรุ่น ผู้ปกครองควรปฏิบัติอย่างไรมากที่สุด

A. แอบดูโทรศัพท์โดยไม่ให้วัยรุ่นเห็น

B. หมั่นขอดูโทรศัพท์ว่าวัยรุ่นคุยกับใคร

C. ชวนคุยเรื่องสื่อออนไลน์เพื่อให้รู้เท่าทัน

D. ให้อิสระวัยรุ่นในการใช้สื่อสังคมออนไลน์

E. ให้วัยรุ่นมาขออนุญาตก่อนเผยแพร่ข้อมูลใดๆ

**Questionnaire S2: *Child and Adolescent parenting Knowledge Evaluation (CAKE*) (English)**

This questionnaire contains 20 items. Please read each question carefully and select the single response that you consider most appropriate.
_____________________________________________________________________________________________

1. When children enter school age or adolescence, what should parents value and support most?
A. Good academic performance and outstanding activities
B. Caring for appearance and personality
C. Sharing the burden of family members
D. Meeting family expectations
E. Self-discovery through diverse activities

2. What is the main purpose of talking about sex with children?
A. To prevent children from keeping secrets from parents
B. To control children’s sexual behavior
C. To prevent sexual intercourse during school age
D. To help children be cautious with the opposite sex
E. To provide correct and age-appropriate information

3. Your 8-year-old child is anxious, wants to compete in sports but fears failure. What should parents do most?
A. Talk with the child about their worries
B. Encourage activities the child feels confident in instead
C. Let the child decide for themselves
D. Offer a reward if the child agrees to compete
E. Insist that the child competes to overcome their fear

4. How should parents give adolescents independence in decision-making?
A. Let adolescents decide on family problems
B. Set boundaries on which matters they can decide themselves
C. Allow them to decide only on personal matters
D. Require consultation before every decision
E. Parents decide on studies and activities for adolescents

5. You find nude photos/videos of same-sex individuals on your 15-year-old’s device and wonder if they might be attracted to the same sex. What should you do most?
A. Wait for your child to bring up the topic themselves
B. Take them to see a doctor for “sexual deviation”
C. Ask them to delete the photos/videos
D. Confront and scold them about sexual preferences
E. Find an opportunity to talk about their sexual interests

6. When you learn that your 9-year-old is being bullied (teased, pushed, or threatened for belongings) at school, what should you do first?
A. Tell the child to deal with it themselves
B. Teach the child to ignore the bullying
C. Teach them to endure teasing patiently
D. Meet with the teacher to deal with the bullies
E. Talk with the child about prevention and coping strategies

7. Your 10-year-old child is overweight to the point of having difficulty moving. What should parents do most?
A. Constantly point out the disadvantages of their weight
B. Restrict sweets and encourage exercise
C. Let the child learn about health risks by themselves
D. Take them to see a doctor about weight
E. Involve the child in adjusting diet and activities

8. Over the past 2 weeks, your 13-year-old isolates in their room, plays games more than usual, is irritable and angry, and cannot concentrate on studying, leading to declining grades. What should parents do most?
A. Warn the child to improve
B. Set a schedule for gaming and bedtime
C. Reduce game time until grades improve
D. Keep the bedroom door open to monitor the child
E. Ask about changes in mood and behavior

9. You find scars on your teenager that suggest self-cutting. What should you do first?
A. Question until the child admits
B. Ensure safety and keep them constantly supervised
C. Express sadness about their actions
D. Ask about the situation and give them time to share
E. Teach them that self-harm is inappropriate

10. Your 15-year-old often skips school, fails several subjects, and fights with peers. What should parents do most?
A. Talk about the negative consequences of behavior
B. Let them experience the consequences on their own
C. Impose punishments for violence
D. Discuss their attitude toward peers and behavior
E. Tell them to stop associating with their current friends

11. In disciplining children, what should parents focus on most?
A. Using harsh punishment so they feel guilty
B. Eliminating behavioral problems completely
C. Teaching them to control emotions and feelings
D. Ensuring obedience to parents’ instructions
E. Promoting children’s self-control skills

12, Your 8-year-old often asks for more time when it’s time to turn off the computer. What should parents do most?
A. Turn it off immediately when time is up
B. Cancel weekend outings
C. Take away the computer and forbid further use
D. Disallow further play and assign other activities
E. Agree in advance on consequences if they exceed time

13. Your 11-year-old argues, shouts, and swears when upset. At that moment, what should parents do most?
A. Tell the child to stop immediately
B. Explain until the child understands
C. Warn them that the behavior is inappropriate
D. Remind them not to speak that way to adults
E. Ask them to calm down first before continuing the talk

14. Your 12-year-old wakes up late, takes too long to shower, dress, and eat breakfast, causing frequent lateness to school. What should parents do most?
A. Wake them up 15–30 minutes earlier
B. Increase punishment for future lateness
C. Create new rules and agreed-upon consequences
D. Constantly urge them to hurry
E. Agree on a departure time and reward for success

15. Your 17-year-old asks to attend a late-night party at a friend’s house and stay overnight. You worry about alcohol. What should you do most?
A. Allow attendance but forbid alcohol
B. Express concerns and forbid going to the party
C. Say you’ll punish them if you find they drank
D. Discuss risks that may come from drinking
E. Offer alternative activities you deem appropriate

16. Your 13-year-old, angry at their younger sister for interrupting gaming, hits her and says it was “like in the game.” What should you do most?
A. Punish the older child for being violent
B. Restrict which games the child can play
C. Ban gaming and assign alternative activities
D. Discuss what is or isn’t appropriate in games
E. Allow media only under parental supervision

17. What is the most appropriate way to manage a 7-year-old’s media use?
A. Set daily screen time limits
B. Allow only educational programs
C. Allow entertainment only on weekends
D. Let the child freely choose what to watch
E. Give extra screen time for finishing homework early

18. How should parents model media use for their children?
A. Watch news alone in the bedroom
B. Watch TV during meals together
C. Change channels immediately when violence appears
D. Critique inappropriate behaviors shown on TV
E. Balance screen time with shared activities with children

19. In managing school-aged children’s social media use, what should parents do most?
A. Allow use only under parental supervision
B. Require children to share the same account as parents
C. Scold when they view inappropriate content
D. Forbid social media before age 12
E. Talk about appropriate and inappropriate online behavior

20. In managing adolescents’ social media use, what should parents do most?
A. Secretly check their phone
B. Regularly ask to see who they are messaging
C. Discuss online media to build awareness
D. Give adolescents freedom to use social media
E. Require permission before posting any information

| **Table S1** preferred sources of parenting information (n = 445) | |
| --- | --- |
| Sources | n (%) |
| Health professionals | 372 (83.60%) |
| Private and public hospitals | 41 (9.21%) |
| Parents and influencers | 18 (4.04%) |
| Governmental institutions | 14 (3.15%) |

Table S1 presents the preferred sources of parenting information as reported by the participants. The majority of respondents (83.60%) identified health professionals as their preferred source of parenting information, reflecting a strong reliance on expert advice. A smaller proportion of participants obtained information from private and public hospitals (9.21%), followed by parents and influencers (4.04%), and governmental institutions (3.15%).

| **Table S2** Differences between media use for parenting information among parents of school age children (N=262) and parents of teenagers (N=183) | | | | | | | | |
| --- | --- | --- | --- | --- | --- | --- | --- | --- |
|  | | | | | | | **95% Interval** | |
|  | | **Statistic** | **df** | **p** | **Mean difference** | **SE Difference** | **Lower** | **Upper** |
| **MUPI** | PM | 2.17 | 443 | 0.03^*^ | 0.26 | 0.12 | 0.02 | 0.50 |
|  | OM | 3.48 | 443 | <0.001^***^ | 0.40 | 0.11 | 0.17 | 0.63 |
|  | LV | -0.03 | 443 | 0.97 | -0.00 | 0.12 | -0.24 | 0.23 |
|  | SV | -0.22 | 443 | 0.81 | -0.03 | 0.15 | -0.33 | 0.26 |
|  | OL | -0.62 | 443 | 0.53 | -0.07 | 0.12 | -0.32 | 0.16 |
|  | QO | 0.86 | 443 | 0.38 | 0.08 | 0.10 | -0.11 | 0.28 |
|  | OT | -0.46 | 443 | 0.63 | -0.02 | 0.05 | -0.13 | 0.08 |
|  | PA | 0.05 | 443 | 0.95 | 0.00 | 0.30 | -0.10 | 0.11 |
| **Parenting** |  | | | | | | | |
| Knowledge | CAKE | 0.31 | 443 | 0.74 | 0.09 | 0.30 | -0.49 | 0.68 |
| Attitudes | PSOC | 0.62 | 443 | 0.53 | 0.62 | 1.01 | -1.36 | 2.61 |
| Practices | PP | 3.08 | 443 | 0.002^**^ | 0.32 | 0.29 | 0.33 | 1.50 |
|  | PI | 3.52 | 443 | <0.001^***^ | 1.63 | 0.46 | 0.72 | 2.54 |
|  | PPM | -6.48 | 443 | <0.001^***^ | -2.96 | 0.45 | -3.86 | -2.06 |
|  | ID | 1.31 | 443 | 0.18 | 0.45 | 0.34 | -0.22 | 1.14 |
|  | CP | 0.98 | 443 | 0.32 | 0.17 | 0.17 | -0.17 | 0.51 |
| Abbreviations: PM = printed materials, OM = online materials, LV = long video clips, SV = short video clips, OL= online listening, QO = questions on social media, OT = online interactive training, and PA = parenting application, CAKE = Child and Adolescent parenting Knowledge Evaluation, PSOC = parenting sense of competence, PP = positive parenting practices, PI = parental involvement, PPM = poor parental monitoring/supervision, ID = inconsistent discipline and CP= corporal punishment; * p < 0.05. ** p < 0.01. *** p < 0.001. | | | | | | | | |

| **Table S3.** Linear regression analysis of media use for parenting information (MUPI) on parenting knowledge, attitudes and practices among parents of school-age children  (N=262) and parents of teenagers (N=183) | | | | | | | |
| --- | --- | --- | --- | --- | --- | --- | --- |
|  | **Parenting Knowledge** | **Parenting Attitudes** | **Parenting Practices** | | | | |
|  | **CAKE** | **PSOC** | **PP** | **PI** | **PPM** | **ID** | **CP** |
|  | **ß (95% CI)** | **ß (95% CI)** | **ß (95% CI)** | **ß (95% CI)** | **ß (95% CI)** | **ß (95% CI)** | **ß (95% CI)** |
| **Model A** | | | | | | | |
| PM | 0.32 (0.02-0.62)^*^ | 1.59 (0.60-2.58)^**^ | 0.09 (-0.21-0.39) | 0.40 (-0.07-0.88) | -0.41(-0.81- -0.01)^*^ | -0.27 (-0.63-0.07) | -0.09 (-0.28-0.09) |
| OM | 0.29 (-0.05-0.64) | 0.75 (-0.38-1.88) | 0.41 (0.06-0.75)^*^ | 0.54 (-0.00-1.09) | -0.55 (-1.01- -0.09)^*^ | 0.00 (-0.39-0.41) | -0.14 (-0.35-0.07) |
| LV | -0.07 (-0.44-0.29) | -1.24(-2.43--0.04) | -0.19 (-0.56-0.17) | -0.23 (-0.81-0.35) | 0.53 (0.04- -1.02)^*^ | 0.08 (-0.34-0.51) | 0.08 (-0.14-0.31) |
| QO | -0.03 (-0.43-0.36) | -0.07 (-1.36-1.22) | 0.36 (-0.02-0.76) | 0.75 (0.12-1.37)^*^ | 0.24 (-2.80-0.76) | 0.42 (-0.98-0.75) | 0.25 (0.00-0.50)^*^ |
| R² | 0.17 | 0.15 | 0.17 | 0.16 | 0.23 | 0.12 | 0.14 |
| **Model B** | | | | | | | |
| PM | -0.03 (-0.40-0.33)^**^ | 0.74 (-0.68-2.16) | 0.27 (-0.12-0.66) | 0.49 (-0.09-1.08) | 0.40 (-0.29-1.09) | 0.21 (-0.25-0.67) | -0.07 (-0.28-0.14) |
| OM | 0.62 (0.22-1.01) | -0.06 (-1.60-1.47) | 0.23 (-0.19-0.65) | 0.33 (-0.29-0.97) | -0.25 (-1.00-0.50) | 0.01 (-0.48-0.51) | -0.04 (-0.27-0.19) |
| SV | -0.39 (-0.73--0.05)^*^ | -1.19 (-2.50-0.11) | -0.17 (-0.54-0.18) | -0.04 (-0.58-0.49) | 0.47 (-0.16-1.11) | 0.53 (0.11-0.96)^*^ | -0.01 (-0.21-0.18) |
| QO | 0.16 (-0.31-0.63) | 1.46 (-0.36-3.29) | 0.07 (-0.43-0.58) | 0.03 (-0.72-0.78) | -0.66 (1.56-0.23) | -0.80 (-1.40--0.21)^**^ | -0.15 (-0.43-0.12) |
| R² | 0.22 | 0.10 | 0.09 | 0.12 | 0.06 | 0.15 | 0.06 |
| Model A represents the regression analysis of parents of school-age children (N=262) with controlled characteristics of study participants, including parent sex, parent age, education, employment status, partnerships status, financial burden, number of children and having a child with health problems. Model B represents the regression analysis of parents of teenagers (N=183) with controlled characteristics of study participants, including education, employment status, family structure, financial burden and number of children. Abbreviations: PM = printed materials, OM = online materials, LV = long video clips. QO = questions on social media, SV = short videos, CAKE = Child and Adolescent parenting Knowledge Evaluation, PSOC = parenting sense of competence, PP = positive parenting practices, PI = parental involvement, PPM = poor parental monitoring/supervision, ID = inconsistent discipline and CP= corporal punishment; * p < 0.05. ** p < 0.01. *** p < 0.001. | | | | | | | |
